# Biocompatible High-Resolution 3D-Printed Microfluidic Devices: Integrated Cell Chemotaxis Demonstration

**DOI:** 10.3390/mi14081589

**Published:** 2023-08-12

**Authors:** Mawla Boaks, Connor Roper, Matthew Viglione, Kent Hooper, Adam T. Woolley, Kenneth A. Christensen, Gregory P. Nordin

**Affiliations:** 1 Department of Electrical and Computer Engineering, Brigham Young University, Provo, UT 84602, USA; 2 Department of Chemistry and Biochemistry, Brigham Young University, Provo, UT 84602, USA; 3 Department of Mechanical Engineering, Brigham Young University, Provo, UT 84602, USA

**Keywords:** microfluidics, 3D printing, concentration gradient, chemotaxis, integrated chemotaxis, biocompatible

## Abstract

We demonstrate a method to effectively 3D print microfluidic devices with high-resolution features using a biocompatible resin based on avobenzone as the UV absorber. Our method relies on spectrally shaping the 3D printer source spectrum so that it is fully overlapped by avobenzone’s absorption spectrum. Complete overlap is essential to effectively limit the optical penetration depth, which is required to achieve high out-of-plane resolution. We demonstrate the high resolution in practice by 3D printing 15 μm square pillars in a microfluidic chamber, where the pillars are separated by 7.7 μm and are printed with 5 μm layers. Furthermore, we show reliable membrane valves and pumps using the biocompatible resin. Valves are tested to 1,000,000 actuations with no observable degradation in performance. Finally, we create a concentration gradient generation (CG) component and utilize it in two device designs for cell chemotaxis studies. The first design relies on an external dual syringe pump to generate source and sink flows to supply the CG channel, while the second is a complete integrated device incorporating on-chip pumps, valves, and reservoirs. Both device types are seeded with adherent cells that are subjected to a chemoattractant CG, and both show clear evidence of chemotactic cellular migration. Moreover, the integrated device demonstrates cellular migration comparable to the external syringe pump device. This demonstration illustrates the effectiveness of our integrated chemotactic assay approach and high-resolution biocompatible resin 3D printing fabrication process. In addition, our 3D printing process has been tuned for rapid fabrication, as printing times for the two device designs are, respectively, 8 and 15 min.

## 1. Introduction

Three-dimensional printing has emerged as an attractive alternative [1,2,3,4] to traditional polymer microfluidic device fabrication techniques such as soft lithography, hot embossing, injection molding, and micro-milling [5,6]. Its appeal is driven in part by capabilities for rapid prototyping, device miniaturization, printing complex structures with high resolution, component integration, and ease of use. Moreover, unlike conventional microfluidic device fabrication technologies, 3D printing can utilize all three dimensions of the device volume for component geometry, placement, and interconnect routing [7,8,9,10,11]. Our group has developed several custom 3D printers and tools based on Digital Light Processing Stereolithography (DLP-SLA), along with optimized resins, to facilitate printing high-resolution microfluidic devices [12,13,14,15,16,17]. Furthermore, we have generalized the 3D printing process to increase the accessible spatial dose parameter space to enable the fabrication of extremely small active devices [10,17]. Examples include membrane valves as small as 45 μm in diameter, a new squeeze valve design with active area down to 15 μm × 15 μm, and an integrated 10-stage two-fold serial diluter with 20 integrated pumps and a footprint of only 2.2 mm × 1.1 mm [17].

As 3D printing for microfluidics has advanced, interest has grown in developing biocompatible materials that are well matched to the capabilities of such 3D printing methods [18,19,20,21,22]. Non-cytotoxic resins suitable for 3D printing high-resolution microfluidic structures have great potential for a wide variety of cell-based applications such as stem cell analysis [23,24], tissue engineering [25,26,27], and the investigation of cell–drug interactions [28,29]. We have previously demonstrated a custom resin for DLP-SLA 3D printing that is effectively non-cytotoxic and is based on avobenzone as the UV absorber [30]. However, in our demonstration we did not address the feasibility of high-resolution 3D printing. In this paper, we show how to achieve high-resolution 3D printing, which involves spectrally filtering the 3D printer optical source so that its emission bandwidth is narrower than and fully overlapped by the avobenzone absorption spectrum. We then demonstrate high-resolution features (15 μm square pillars with 7.7 μm gaps), followed by membrane valves and pumps that are extremely robust (1,000,000 valve actuations). Next, we develop a concentration gradient (CG) generation module that includes pillar arrays and embed this module in two chip designs. The first design includes only chip-to-world interconnects to facilitate CG generation with off-chip syringe pumps. In contrast, the second incorporates on-chip valves, pumps, and reservoirs to create a fully integrated design. We then apply devices of each type to a study of adherent cell chemotaxis in the presence of a chemoattractant CG and show that both designs are equally effective for determining cell motility in comparison to control runs in the absence of a CG. Our results show that high-resolution 3D printing of biocompatible and fully integrated devices incorporating active and passive components is now possible.

## 2. Materials and Methods

### 2.1. 3D Printer and Materials

For all 3D printing reported in this paper, we used a third-generation custom 3D printer with a 365 nm LED light source as described in Ref. [10]. The projected image has a pixel pitch (x-y resolution) of 7.6 μm. The x-y-z build volume is 19.46 mm × 12.16 mm × 30 mm, respectively. The resin consists of poly(ethylene glycol) diacrylate (PEGDA, MW 250) with 1% (*w*/*w*) phenylbis(2,4,6-trimethylbenzoyl)phosphine oxide (Irgacure 819) photoinitiator and 0.38% (*w*/*w*) avobenzone UV absorber. Hereinafter, we refer to this as A-PEGDA resin. PEGDA and Irgacure 819 were purchased from Sigma-Aldrich (St. Louis, MO, USA), whereas avobenzone was ordered from Fisher Scientific (Waltham, MA, USA). All materials were used as received.

### 2.2. Optical Absorbance Measurement

To measure the absorption spectrum of the UV absorber, we mixed 0.06% *w*/*w* avobenzone with PEGDA and infused the resultant solution between two glass slides separated by a 120 μm spacer. We used attenuated light from a broadband XCITE-120Q source (Lumen Dynamics, Mississauga, ON, Canada) to illuminate the resin through one of the glass slides and captured the transmitted light using a fiber with a 100 μm core and connected to a QE65PRO spectrometer (Ocean Optics, Dunedin, FL, USA). We also performed the same measurement except with only PEGDA between the glass slides, which gave a baseline transmitted spectrum without avobenzone but with the effects of the glass slides, PEGDA, and surface reflections on the incident source spectrum. We used this baseline spectrum with the known resin thickness (120 μm) and measured the spectrum with avobenzone to calculate the molar absorptivity of the absorber. The 3D printer optical source spectrum was measured with the same fiber and spectrometer.

### 2.3. Polymerization Thickness as a Function of Exposure Time

Polymerization thickness as a function of optical exposure time was measured by applying a thin layer (~1 mm) of resin on the 3 × 4 inch${}^{2}$ glass plate that comprises the bottom of the 3D printer resin tray. The printer focus was adjusted so the projected image was focused on the top surface of the glass plate. Next, the resin was exposed to a series of patterns with different exposure times at different locations in the projected image region. This resulted in different polymerization thicknesses at those locations. The glass plate was then removed from the resin tray and rinsed with isopropyl alcohol (IPA) to remove unpolymerized resin. Finally, the thicknesses of the 3D-printed patterns were measured with an Alpha-step 200 profilometer (Tencor Instruments, Milpitas, CA, USA).

### 2.4. Three-Dimensional Printing

Microfluidic devices were 3D-printed on 25 mm square silanized glass slides. Each slide was first cleaned with acetone and IPA. Next, slides were immersed in a solution of 10% 3-(trimethoxysilyl)propyl methacrylate and 90% toluene for 2 h. After silane deposition, the glass slides were stored in fresh toluene in a closed container until use. Unless otherwise noted, all 3D prints reported in this paper were fabricated with 10 μm thick layers. The measured image plane irradiance was 35.25 mW·cm${}^{-2}$.

### 2.5. Device Fabrication

Active devices reported in this paper used 150 μm diameter membrane valves [7,17,31]. Each valve consists of two cylindrical voids of different heights separated by a 5 μm thick membrane, all 3D-printed with A-PEGDA resin. The top cylinder (control chamber) height is 40 μm, while that of the bottom cylinder (fluid chamber) is 20 μm. Only the membrane layer was printed with a 5 μm thickness. It received a 175 ms exposure time, while the 10 μm layers in the print had a 240 ms exposure time. As noted in Ref. [17], the ability to control all aspects of 3D printer operation, including individual layer thickness and exposure time, is a critical enabling feature of our 3D printing approach for microfluidics. The motivation for reduced membrane layer thickness is increased membrane deflection for a given actuation pressure in the control chamber (17.25 psi for valve lifetime measurements and 20 psi for flow rate measurements) to ensure sufficient deflection of the membrane into the fluid chamber to block the central inlet channel, which constitutes valve closure.

After 3D printing, unpolymerized resin was initially cleared from each device by manually applying vacuum to each pneumatic and fluidic port, followed by carefully flushing the interior of the valves and the flow channels with IPA. Another round of vacuum was then applied to extract residual IPA from the devices.

Next, the devices were optically cured by placing it in a custom curing station for 20 min. The curing station has a 430 nm LED (Thorlabs, Newton, NJ, USA) that generates a measured irradiance of 11.3 mW/cm${}^{2}$ in the curing plane. Note that the LED wavelength was carefully chosen so that much of the LED’s emission spectrum was outside of the avobenzone absorption spectrum such that the curing light could penetrate deeply into the devices while still being within the absorption spectrum of the photoinitiator to enable further polymerization during curing.

Before use, chip-to-world interfaces were created by inserting Microbore PTFE tubing (0.022${}^{\prime \prime }$ID × 0.042${}^{\prime \prime }$OD) into corresponding cylindrical holes printed in the devices. A UV-curable epoxy (DecorRom, Amazon, Seattle, WA, USA) was used to glue the tubing in place and to plug the end of each flushing channel. Flushing channels were designed to terminate at the side of the chip near the tubing holes to facilitate convenient plugging with the UV epoxy. The use of UV-curable epoxy dramatically reduces the tubing attachment time to just a few minutes from over 2 h as reported in our previous work [7].

### 2.6. Concentration Gradient Verification

Devices with a CG module for cellular migration contain source and sink channels to continuously supply and remove, respectively, chemoattractant [32,33]. The CG magnitude, uniformity, and stability were characterized using a fluorescein-containing solution as the source and an identical solution without fluorescein as the sink. For syringe pump devices, the source channel was loaded with 40 μM fluorescein in water and the sink channel with only water. After one hour of flow at 64 μL/h, images were captured every ten minutes for three hours using an FITC filter in a fluorescence microscope (Olympus IX73 with Hamamatsu ORCA-Flash4.0 C11440 camera, Hamamatsu Photonics Deutschland GmbH, Herrsching, Germany).

Devices containing on-chip pumps were primed with Mili-Q water before adding fluorescein. An amount of 130 μM fluorescein was used to verify gradient stability in devices powered by on-chip pumps. A phase interval of 75 ms between actuations was used, which resulted in a 375 ms pumping period and delivered an estimated 54 μL/h of fluid to each of the source and sink channels.

Before every cell chemotaxis run for both device types, a similar fluorescein CG check was performed to ensure the presence of the expected CG. These were run at 64 and 65 μL/hr for the syringe pump and integrated device designs, respectively.

### 2.7. Cell Migration

To prepare devices for use with cells, post-print optically cured 3D-printed devices were baked in a dry oven at 80 ${}^{\circ }$C for four days, which helps improve cell adhesion and biocompatibility as shown in Supplementary Information Section S1. We note that further investigation is warranted to determine the specific cause of adhesion and biocompatibility improvement. For the present study, experimental evidence of improvement is sufficient.

Following baking, PTFE tubing was glued into inlets and outlets using UV resin glue. Before use, each device was flushed with 70% ethanol for sterilization followed by coating with collagen to promote cell adhesion since cell adhesion is otherwise weak [30]. Collagen coating involved filling channels with 0.2 mg/mL Type I collagen overnight at room temperature and removing the liquid with a vacuum before use.

At the beginning of an experiment, devices, fluids, and syringes were heated to 37 ${}^{\circ }$C using a stage heater and climate-controlled microscope box as a bubble prevention measure by reducing temperature-dependent gas solubility in the fluid. Once the temperature equilibrated, devices were primed using a cell seeding solution comprising 10% fetal bovine serum (FBS), 4.5 g/L D-glucose, and Pen-Strep in phosphate-buffered saline (PBS). Human lung fibroblasts (HLF1s, ATCC, USA) were added to primed devices with a seeding density of $2\times 10^{6}$ cells per mL, which resulted in 25 to 60 fibroblasts in a CG channel. Fibroblasts were allowed two minutes to attach to the collagen-coated polymer without flow and an hour to fully spread with a flow rate of 64 μL/h.

In syringe pump devices after cell seeding, the cell seeding solutions in the syringes were replaced with fibronectin and serum-free solutions to prepare for the chemotaxis assay. The source and sink channels contained filter-sterilized 1% BSA with 4.5 g/L D-glucose and Pen-Strep in PBS. However, the source channel also contained 10 μg/mL Human fibronectin (hFN) as a chemoattractant [34,35].

To record cell movement, we acquired a brightfield time-lapse video using a 10× objective lens for about 4 h. Cell motility was analyzed using the ImageJ Manual Tracking and Ibidi Chemotaxis Tools plugins [36]. In control runs, there was no CG as cells were treated with uniform concentrations of the seeding solutions in the source and sink channels.

The same procedure was used to prepare integrated devices with on-chip pumps and to conduct each chemotaxis assay. After cells attached to the surface of the migration chamber, the pumps were turned on with an initial phase interval of 120 ms. Over 3 to 5 min, the pump phase interval was incrementally decreased to 60 ms to reach a final average volumetric flow rate of 65 μL/h for each pump. The source and sink wells on each chip were refilled every 40 min throughout the assay.

## 3. Results and Discussion

In this section, we first illustrate our spectral engineering approach, which is necessary to achieve the highest z resolution, followed by a general resolution demonstration in the form of an array of pillars, and then 3D-printed valve lifetime testing, pump characterization, and cell chemotaxis measurement with the 3D-printed devices that make use of pillar arrays, valves, and pumps.

### 3.1. Three-Dimensional Printer Spectrum Tailoring

As we showed in Refs. [12,37], z resolution is fundamentally a function of the optical penetration depth, $h_{a}$, which is the depth at which the irradiance drops to 1/e of its initial value, rather than the layer thickness used during 3D printing. Given $h_{a}$ for a particular resin, we have established a rule of thumb for the choice of layer thickness, namely, 0.77 $h_{a}$, to best achieve the highest z resolution, which can be defined as the minimum achievable height for a closed channel. We have empirically found this height to be 2.3 $h_{a}$ [12]. If the intended minimum closed channel height for a particular design is significantly greater than 2.3 $h_{a}$, the layer thickness choice can often be relaxed from 0.77 $h_{a}$ to be in the range of 0.5 $h_{a}$ to $h_{a}$ [37].

The optical penetration depth must be reduced to increase the 3D printing z resolution for microfluidic devices. This is achieved by increasing the resin’s absorption of the light projected by the 3D printer, which is typically accomplished by including a soluble UV absorber in the resin and increasing the absorber’s concentration [12,37].

**Figure 1:**
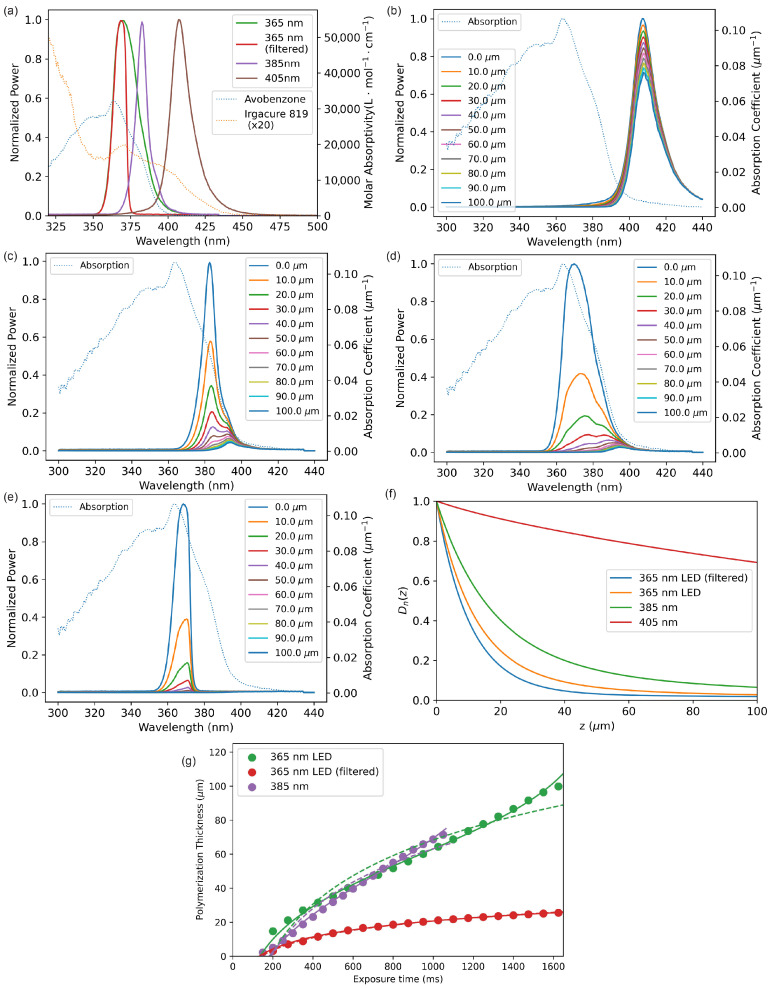
(**a**) Measured molar absorptivities and LED source spectra. Note that the Irgacure 819 absorption spectrum is scaled by a factor of 20 to be visible in the molar absorptivity range covered by the right axis. (**b**–**e**) Evolution of source spectrum for successive propagation distances in 0.38% avobenzone and 1% Irgacure 819 PEGDA resin for (**b**) 405 nm, (**c**) 385 nm, (**d**) 365 nm, and (**e**) short-pass-filtered 365 nm LED sources. (**f**) Normalized dose for each LED source as a function of polymerization depth. (**g**) Measured thickness (circles) as a function of exposure time, along with curve fits to Models 3 (dashed lines) and 4 (solid lines) from Ref. [12]. For the filtered 365 nm LED, both curve fits lie on top of each other and overlap the measured data because there is good spectral overlap between the incident light and the resin’s absorption. The difference between the fits for the other cases represents incomplete spectral overlap.

An underappreciated fact in the 3D-printed microfluidics community is that it is also essential for the resin’s absorption spectrum to span the source emission spectrum fully. For example, consider Figure 1a, which shows the measured molar absorptivity of avobenzone in PEGDA (dashed blue curve) and 1% Irgacure-819 in PEGDA (dashed orange curve scaled by a factor of 20 to make it visible) compared to the measured emission spectra for 405 nm, 385 nm, 365 nm, and short-pass-filtered 365 nm LEDs used in our custom 3D DLP-SLA printers. Note that the long wavelength cutoff of the avobenzone UV absorber is ∼400 nm, while the photoinitiator (Irgacure 819) is sensitive up to ∼440 nm. This means that during printing, source light between 400 and 440 nm can penetrate deeply into previously printed layers, limited only by the small absorption of the photoinitiator. Absorption by the photoinitiator causes further polymerization in these deeper regions, leading to undesired polymerization of entrapped unpolymerized resin in the already printed layers, which reduces the achievable z resolution of negative space features.

To illustrate this concept, consider A-PEGDA resin in combination with a 405 nm LED source. Figure 1b shows the calculated light spectrum at 10 μm depth intervals as the light propagates through the resin. Even at a resin depth of 100 μm (i.e., ten 10 μm layers), most of the light is still transmitted because the source spectrum is largely outside of the absorption spectrum of the resin, which is dominated by the avobenzone UV absorber spectrum. This is further illustrated in Figure 1f, which shows the normalized dose as a function of propagation distance, *z*, in the resin, where the normalized dose is defined as the energy per unit area at a depth *z* divided by the energy per unit area at z=0 [12]. For the 405 nm source, the dose at 100 μm is 69% of the dose at z=0μm. Therefore, the absorber/LED spectrum mismatch makes it impossible to obtain high-resolution voids in the z dimension with this resin and optical source.

As illustrated in Figure 1c, the situation is substantially improved with a 385 nm LED. However, there is still not enough absorption of the long wavelength tail of the LED emission to enable the highest possible resolution in z. As shown in Figure 1f, the normalized dose at 100 μm is still 7%. Use of a 365 nm LED (Figure 1d) yields further improvement, but not as much as one might expect because the 365 nm LED spectrum has an extended long wavelength tail that matches the 385 nm LED long wavelength tail for wavelengths greater than 400 nm. From Figure 1f, the normalized dose at 100 μm is 3%. For the limited number of 365 nm LEDs that we have measured, we have found that all exhibit this undesirable extended long wavelength tail.

An obvious solution [38] is to use a UV short-pass filter to eliminate the 365 nm long wavelength tail. Figure 1e shows the evolution of the spectral power of a 365 nm LED paired with a custom 370 nm UV short-pass filter. Note that the full spectral width of the filtered light lies entirely within the high absorption portion of the avobenzone spectrum, yielding effective reduction in the optical power across all wavelengths of the filtered spectrum. This is the condition required to obtain the highest z resolution and illustrates that the spectral properties of the resin and LED should be designed in conjunction with each other, i.e., they cannot be chosen independently.

The dramatic effect of good spectral coverage limiting optical penetration is illustrated in Figure 1g, which shows measured polymerization thickness as a function of exposure time. Note that for the 365 and 385 nm LED data, the polymerization thickness increases similarly for exposure times greater than ∼300 ms because, as previously noted, their emission spectrum long wavelength tails are alike. Moreover, in both cases the polymerization thickness increases almost linearly with exposure time, demonstrating the deleterious effect of a lack of absorption of the long wavelength tails. On the other hand, the filtered 365 nm LED data show a logarithmic dependence of polymerization thickness on exposure time, which is expected for the case of good source/absorber spectral overlap as discussed in detail in Ref. [12], and which is required to obtain the highest z resolution.

In our earlier publications [12,37], we ensured that we could meet the condition of good spectral overlap by deliberately choosing UV absorbers with broad enough spectral absorption to fully cover our 3D printer source emission spectrum. Since the long wavelength tail of the LED emission is in the blue part of the visible spectrum, such absorbers had to absorb in this spectral region which resulted in a yellowish (NPS absorber) [12] or orange (Sudan I absorber) [37] coloring of 3D-printed parts. In this paper, we demonstrate modifying the source emission spectrum to enable the use of a UV absorber (avobenzone) that has otherwise desirable properties, i.e., biocompatibility [30] and full transparency across the visible spectrum. Modifying the source spectrum via filtering reduces the total optical power reaching the resin. We have found that this is a small price to pay to gain the benefit of the highest z resolution because even with filtering, our exposure times tend to be only a few hundred milliseconds, with a total layer-to-layer elapsed fabrication time as short as 1 s, which enables rapid fabrication of devices.

### 3.2. Pillar Array Fabrication

**Figure 2:**
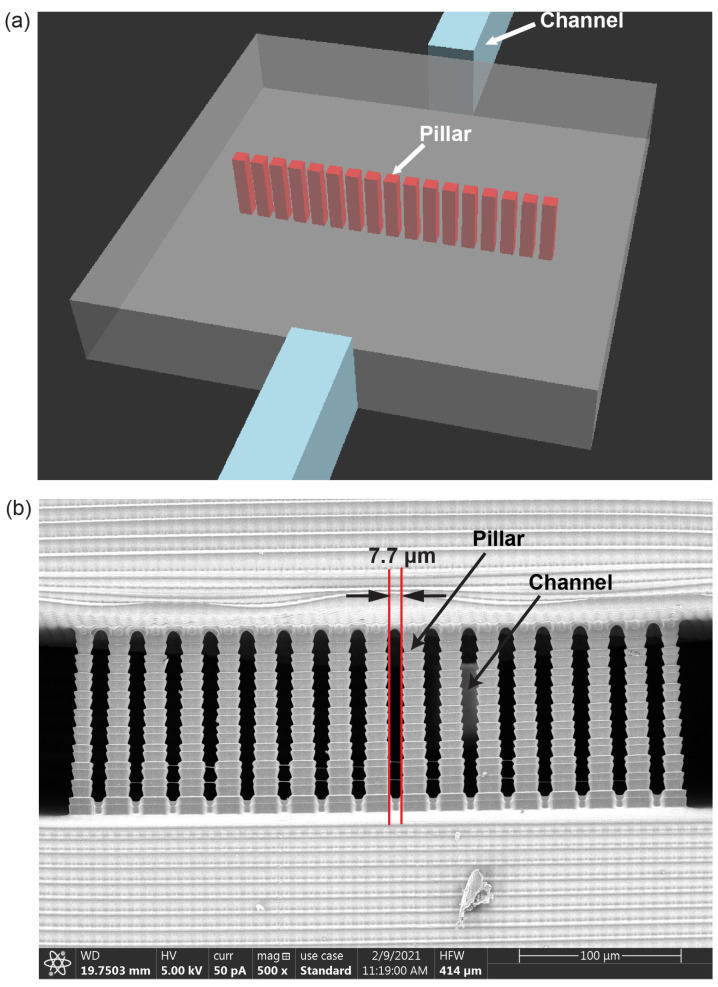
(**a**) Schematic diagram of a pillar array (red) within a chamber, and (**b**) cross-section SEM image of corresponding 3D-printed pillar array.

As a simple demonstration of the high-resolution 3D printing capability of our approach, and in anticipation of using such features in our cell chemotaxis devices, consider the CAD design of a linear array of pillars in a small chamber as shown in Figure 2a. The pillars are 2 × 2 pixels (15.2 × 15.2 μm${}^{2}$) with single-pixel (7.6 μm) gaps spanning the height of the chamber. The chamber is 0.53 mm long and 1.14 mm wide, with a height of 100 μm. Channels with cross-section 60 μm × 100 μm are placed at both ends of the chamber to facilitate flushing unpolymerized resin after 3D printing.

As we have shown [39], sparse positive features and narrow gaps are challenging to 3D print in microfluidic devices. In part, this is due to scatter from the FEP film used on the bottom of a resin tray to reduce adhesion between just-printed layers and the underlying resin tray window. Such scatter represents a feature-dependent background illumination, which is in addition to the intended image illumination that defines the desired photopolymerized pattern for a given layer. Poor image focus compounds the problem, especially for high-resolution features. For example, in our experience, the focus needs adjustment to within ±10 μm of the ideal focus in our system, despite the manufacturer’s ±50 μm depth of focus specification for our imaging optics. Another compounding factor is that pillar-like structures within any void region usually need a higher dose compared to the bulk of the device to achieve the designed dimensions [39]. This makes it even more challenging to print pillars with single-pixel 7.6 μm gaps.

Our solution is to maintain tight printer focus and leverage our 3D printer software’s flexibility to use customized layer thicknesses and optical dose settings for the pillar features. This enables us to achieve excellent dose control in all three dimensions [17]. For the pillar features, we used a 5 μm layer thickness and variable layer exposure time (500 ms for the bottom four pillar layers, 725 ms for the other 16 pillar layers). In comparison, all other features were printed with 10 μm layer thickness and 250 ms exposure time. Note that this entailed printing two 5 μm pillar layers for every 10 μm layer in which they were embedded. The result is shown in the cross-sectional scanning electron microscope (SEM) image in Figure 2b. The measured spacing between the pillars and the measured pillar width are 7.7 and 15.2 μm, respectively, demonstrating that our approach effectively achieves challenging high-resolution features.

### 3.3. Robustness of 3D-Printed Valves

**Figure 3:**
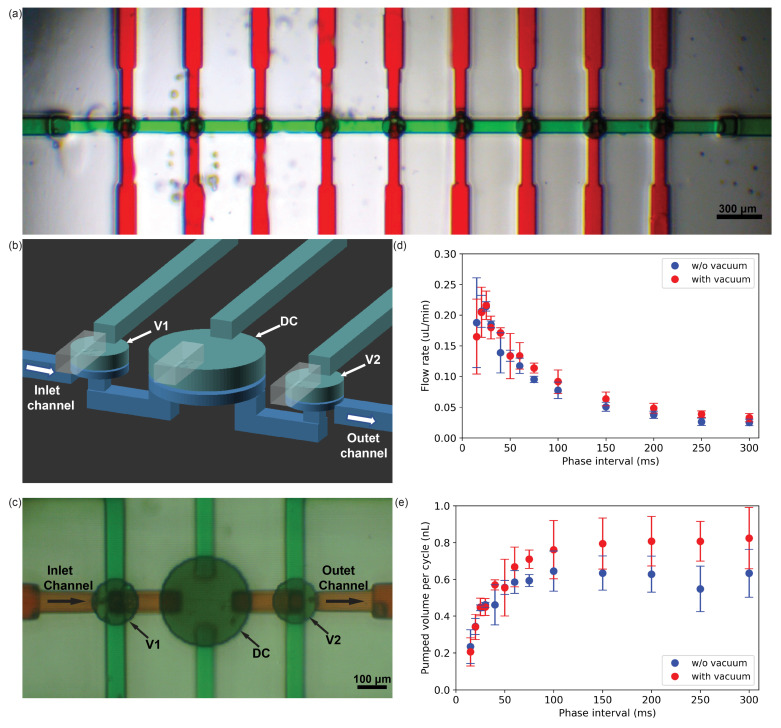
Three-dimensionally printed (**a**) valves and pumps. (**b**) Schematic diagram and (**c**) microscope image of pump with 150 μm diameter valves and 300 μm diameter DC. (**d**) Measured volumetric flow rate and (**e**) volume pumped per cycle as a function of the phase interval. Circles are average values for three tested pumps, and error bars indicate ±1 standard deviation.

In 2018, we demonstrated that a set of five 300 μm valves fabricated with our NPS-PEGDA resin [12] could survive 1 million actuations with no apparent degradation in functionality [13]. In the current study, we conducted a new valve actuation robustness test with our A-PEGDA resin and 150 μm valves. Three devices were tested, each with nine valves as shown in Figure 3a, where, for the purpose of visualization, the pneumatic channel and fluid channels are shown with green- and red-dyed water, respectively. The pneumatic channel was common to all valves so all could be actuated with a single pneumatic input. The pneumatic channel was alternately driven for 30 ms at 17.25 psi, followed by 30 ms at atmospheric pressure to create a repeating valve closed/open sequence. During the test, the fluid channels were connected to a common pressurized water source in the form of a small elevated tank, which resulted in a periodic fluid flow through each valve as the valves were actuated. The valves in each device were subjected to 1 million periodic actuations, which took ~17 h per device. After testing, the valves in each of the devices showed no sign of leakage or degradation, and continued to operate normally, indicating that the valves fabricated with our A-PEGDA resin are quite robust.

### 3.4. Characterization of 3D-Printed Pumps

As shown in Figure 3b, a pump consists of two valves (V1 and V2) that sandwich a valve-like displacement chamber (DC) [7,14,17]. The DC has the same basic structure as a valve except its fluid channels are both located at valve edges so it cannot achieve a closed state and instead functions solely to displace fluid. The five-phase pump actuation cycle that we use is illustrated in Table 1, [7,14,17], where at $t_{0}$ the valves and DC are actuated such that the valves are closed, and the DC fluid chamber is in its minimum fluid volume state. At $t_{1}$, pressure is released or a vacuum is applied to valve V1 and the DC, such that the DC fluid chamber transitions to its maximum fluid volume state by drawing fluid from the inlet channel through V1. At $t_{2}$, V1 is closed, isolating the fluid in the DC. At $t_{3}$, V2 is opened, followed by the DC being actuated at $t_{4}$ to expel its fluid through V2 into the outlet channel. The entire process then repeats for the next pump cycle. With this actuation cycle, the only function performed by the DC is to displace fluid, which is why we do not configure it as a valve with a closed state. The change in DC fluid volume between its actuated and unactuated states defines the maximum fluid volume that can be pumped during each pumping cycle.

The 3D-printed pump in Figure 3 has a 300 μm diameter DC connected to two 150 μm diameter valves. The rest of the pump parameters are summarized in Table 2. The build layer thickness is 10 μm except for the membrane layers. Each valve membrane is printed as one 5 μm layer, whereas the DC membrane is printed as two successive 5 μm layers because it has a larger diameter. The 5 μm thick membrane layers were each exposed for 175 ms. Figure 3c shows a bottom-view microscope photo of a 3D-printed pump where the control and fluid channels are filled with aqueous green and red dye, respectively.

We measured the flow rate and volume pumped per cycle as a function of the phase interval shown in Figure 3d,e, respectively. In the “*w*/*o* vacuum” case, the control chambers switch from positive pressure (closed) to atmospheric pressure (open). In this case, the membrane returns to an un-stretched condition from a stretched condition due solely to elastic strain. In the “with vacuum” case, an applied vacuum is applied instead of atmospheric pressure, which augments the membrane restoration force and additionally pulls the membrane up into the control chamber from the undeflected state. This results in an increased pumped volume per cycle for phase intervals >50 ms. For example, at the 150 ms phase interval, the average pumped volume per cycle increases from 0.63 nL to 0.79 nL. The behavior and performance of pumps fabricated with A-PEGDA resin follow the trends we have observed for pumps fabricated with NPS-PEGDA resin [17].

### 3.5. Chemotaxis Device Development and Fabrication

Cell chemotaxis is a critical physiological process in which cells migrate in response to a chemical gradient such as those formed by growth factors in angiogenesis [40,41] and embryogenesis [42]. Various microfluidic devices have been developed to create concentration gradient generators for cell chemotaxis studies. They typically can be categorized as one of two types. In the first, the CG is set up solely through analyte diffusion, which has the disadvantage of often needing hours to establish a gradient that then continuously changes in time, which can complicate the interpretation of experimental results [43,44]. The second type uses advection to continuously supply source and sink fluids to the CG region [45]. In this region, a gradient is formed using some combination of advective and diffusive mass transport, with the ideal in many cases being dominantly diffusive mass transport. The geometry of the microfluidic device and details of source/sink fluid flow determine the degree of diffusive versus advective mass transport.

**Figure 4:**
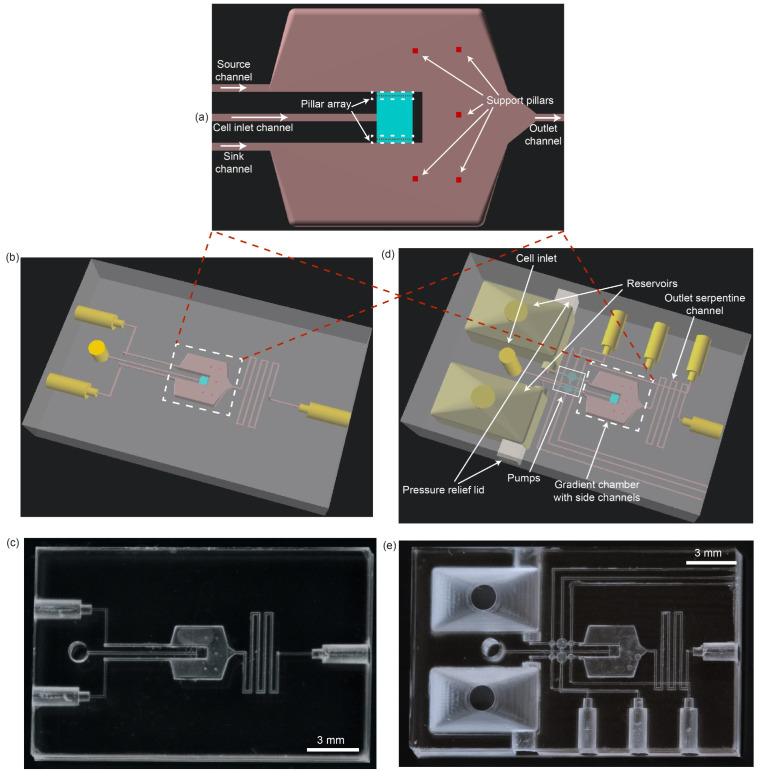
(**a**) Schematic diagram of 100 μm tall concentration gradient channel (light blue) straddled by wide 500 μm tall source and sink channels. (**b**,**c**) Schematic diagram and photograph of 3D-printed device designed for syringe pump operation, respectively. (**d**,**e**) Same except for integrated device design that includes source and sink fluid reservoirs and on-chip pumps.

In Figure 4a, we show our design for a 3D-printed CG region (light blue) in which diffusive mass transport is dominant, and which is supplied by source and sink flows from much larger channels that have slow fluid flow to minimize advective coupling to the much smaller CG channel. The length, width, and height of the CG channel are 715 μm, 500 μm, and 100 μm, respectively, with an array of 15.2 μm pillars inset by 45 μm from each entrance to the channel. This reduces the effective CG channel length to 593 μm. The pillars have 15.2 μm spacing between them. In contrast to the CG channel, the source and sink channels are five times taller (500 μm) and are much wider (1064 μm at the widest part of the channel). The result is that the CG channel is only 0.9% the volume of the source and sink channels with minimal advective coupling to the CG channel.

In our design, a third channel connects to the center of the CG channel (see Figure 4a), through which cells are pipetted into the CG channel. After pipetting, the channel input is sealed with paraffin film to prevent unwanted fluid advection through this channel. The downstream region where the source and sink flows merge before exiting through the outlet channel is so large that we introduced a handful of 76 μm (10 pixel) square pillars to provide support to the roof layer. The support pillars span the entire 500 μm channel height and help avoid warping of the roof during the 3D printing process.

As shown in Figure 4, we developed two 3D-printed CG chips that use the same CG region design. In the first case, Figure 4b,c, the CG region channels (source, sink, cell, and outlet) are connected directly to cylindrical chip-to-world connections to which tubing is attached. This design is used with an off-chip dual syringe pump to provide identical source and sink fluid flows for cell chemotaxis experiments. We refer to this design as the syringe pump chip design. We refer to the second CG case, Figure 4d,e, as the integrated chip design because it includes on-chip reservoirs into which source and sink fluids are pipetted, and two identical on-chip pumps, one for each reservoir, to generate the source and sink fluid flows. Three pneumatic inputs drive the pumps in parallel, meaning that both pump inlet valves share a common pneumatic input, as do both DCs and both outlet valves, thereby ensuring that the pumps operate in sync with each other. Each reservoir has a pressure relief pathway that is open during 3D printing to prevent bursting the inlet valve membranes by providing an alternate resin fluidic path with very low fluidic resistance. After post-print flushing of uncured resin from negative space features (channels, reservoirs, pumps, etc.), 3D-printed lids are glued into these holes to finish each reservoir prior to device use.

CG devices of both types were printed with a bulk dose of 240 ms and 10 μm layer thickness. Both the pillar array separating the CG channel from the source and sink channels and the roof support pillars were printed with 10 μm layers and an optical dose of 700 ms.

Initial experiments with live cells showed that the cells suffered from a lack of nutrients and oxygen at fluid flow rates below 50 μL/h. For the integrated chip design, we therefore scaled up the pump dimensions to those listed in Table 3 to ensure adequate nutrient and oxygen supply to cells in the CG channel. During 3D printing, DC membranes were given an additional secondary dose of 160 ms with 76 μm diameter larger than the actual membrane diameter to toughen the membrane and the anchoring region in the surrounding material as required for larger diameters [14]. The valve membranes were given a dose of 265 ms. Devices were also cured post print for an additional 25 min to further toughen the membranes. Under these conditions, we measured flow rates of 56, 65, and 54 μL/h at 50, 60, and 75 ms phase intervals, respectively, with 40 psi actuation pressure and vacuum. The higher actuation pressure was chosen to accommodate the increased valve and DC membrane thickness and fluid chamber height.

We note another important feature that we implemented for both syringe pump and integrated chip fabrication. When devices are generated with the 3D printer in excellent focus, horizontal (x-y) surfaces look pixelated because gaps between micromirrors in the DMD device are resolved in the projected image and are faithfully replicated in the polymerized material. The result during microscope observation is a readily apparent background pixelation that makes it difficult to automate tracking cell movement over the course of a chemotaxis experiment. To solve this problem, we utilized the capability of our 3D printer to specify an arbitrary amount of defocus for any given projected image during 3D printing. With this capability, we defocused the two CG channel floor layers and the two CG channel roof layers by 106 μm (the equivalent of 14 pixels) and gave them an additional layer exposure time of 700 ms. The result was a significant reduction in background pixelation during microscope observation of the CG channel region, making it much easier to employ automatic cell tracking tools. Example microscope images of as-printed surfaces are shown in Figure S2.

Finally, since 3D printing is a layer-by-layer process, the print time is roughly equal to the product of the number of layers in a print and the average cycle time for a layer. The latter is the sum of the exposure time, the duration of the build platform’s up movement and subsequent down movement, and any intentionally specified wait times. Since the integrated device requires more layers (426 layers compared to 260 layers) to accommodate the additional volume needed by the including reservoirs, it takes 15 minutes to print. In contrast, the syringe pump device requires only 8 minutes to print.

### 3.6. Concentration Gradient

**Figure 5:**
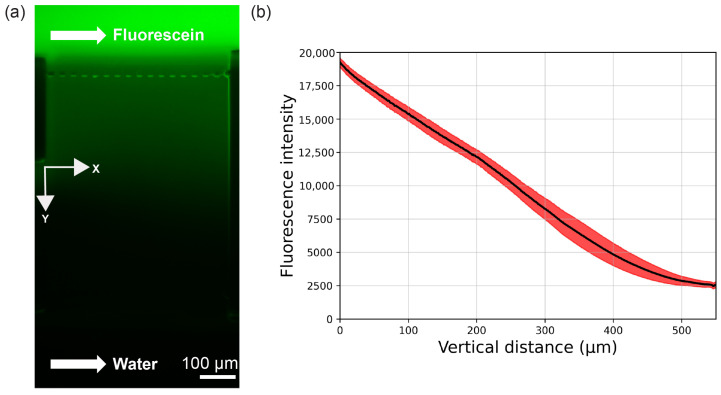
(**a**) Fluorescence microscope image of fluorescein CG between advective source and sink channel flows. (**b**) Average fluorescence intensity (black line) as a function of y. The average is calculated across the width of the channel (x direction) for each y position, and the red band indicates ±1 standard deviation.

A representative CG for an integrated device design is shown in Figure 5a where the gradient is formed between a fluorescent source flow and non-fluorescent sink flow. Figure 5b shows the average fluorescence intensity in the y direction (black line), where the average is taken in the x direction for each y position. The red band indicates ±1 standard deviation of the average value. Note that the CG is reasonably linear in y and uniform in x (i.e., lateral spatial uniformity) and that the fluorescence spans a nearly seven-fold concentration range. An analysis of similar images acquired every 10 minutes over a 3-hour time period yields comparable results, with a concentration range that is six- to seven-fold, indicating acceptable CG temporal stability. Likewise, a comparable analysis of fluorescent CGs generated in syringe pump devices gives similar results for CG linearity, lateral spatial uniformity, and temporal stability.

### 3.7. Syringe Pump HLF1 Chemotaxis

**Figure 6:**
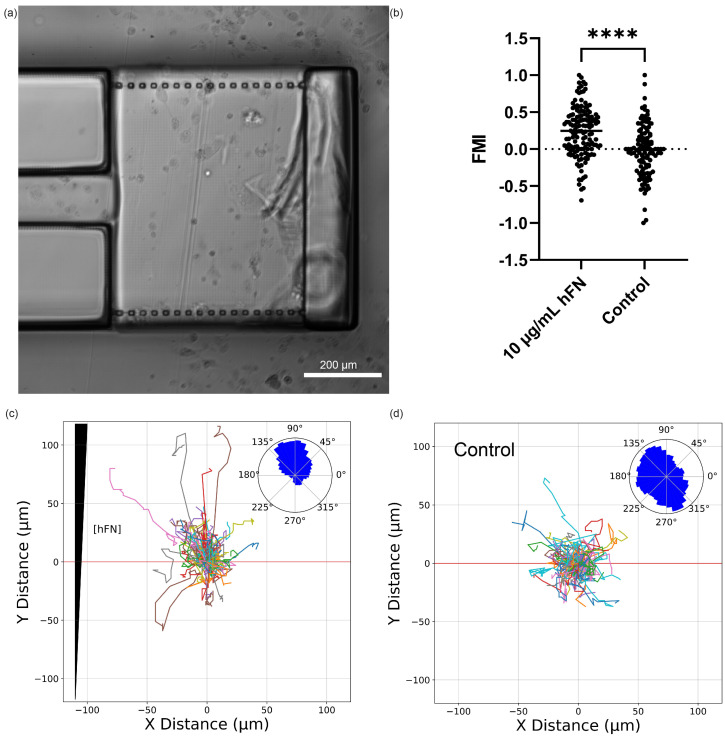
Syringe pump device cell migration: (**a**) Microscope image of gradient migration chamber with adhered HLF1 cells. (**b**) Forward Migration Index (FMI) for experiment and control runs, **** *p* < 0.0001. (**c**,**d**) Track plot of cells from gradient migration and control runs, respectively. Corresponding polar histograms are shown as insets.

Figure 6 summarizes the experimental results of the chemotaxis assay for the syringe pump device design and includes an image of cells seeded in the CG channel (Figure 6a). Chemotaxis was measured using the Forward Migration Index (FMI), defined as [36]

(1) $$FMI=\frac{1}{n}\sum_{i=1}^{n}\frac{y_{i,end}}{d_{i,accum}}$$

in which *n* is the number of cell tracks, $y_{i,end}$ is the y distance at the end of the *i*th track, and $d_{i,accum}$ is the accumulated distance of the *i*th track. Figure 6b compares the FMI for cell migration in an hFN CG versus a no-chemoattractant control. Briefly, HFL1 cells were observed while in an hFN CG (10 μg/mL source concentration) to see whether they would migrate toward the chemoattractant gradient. The source and sink contained the same solution lacking an hFN chemoattractant for control experiments, so there was no CG. Across three biological replicates for the CG experiments (*n* = 127 total tracked cells), we measured an average FMI of 0.24. The no-CG control experiments had a measured averaged FMI of −0.02 (*n* = 104 total tracked cells). The CG experiments showed significant chemotaxis (*p* < 0.0001) towards the hFN CG.

The track plots in Figure 6c,d were generated to help visualize all the cell tracks over the three biological replicates for the CG and no-CG control, respectively. In the presence of the hFN chemoattractant CG, most cells migrated up the chemoattractant gradient (Figure 6c). Additionally, we generated polar histograms (inset of Figure 6c,d) to show the cell counts of directional migration in each polar direction. In the case of Figure 6c, the polar histogram indicates that most HLF1 movement occurred in the direction of the hFN source. The control polar histogram in Figure 6d inset indicates random movement in ±y. The asymmetry of migration in x may be due to small uncharacterized advective flow within the CG channel.

### 3.8. On-Chip Pump HLF1 Chemotaxis

**Figure 7:**
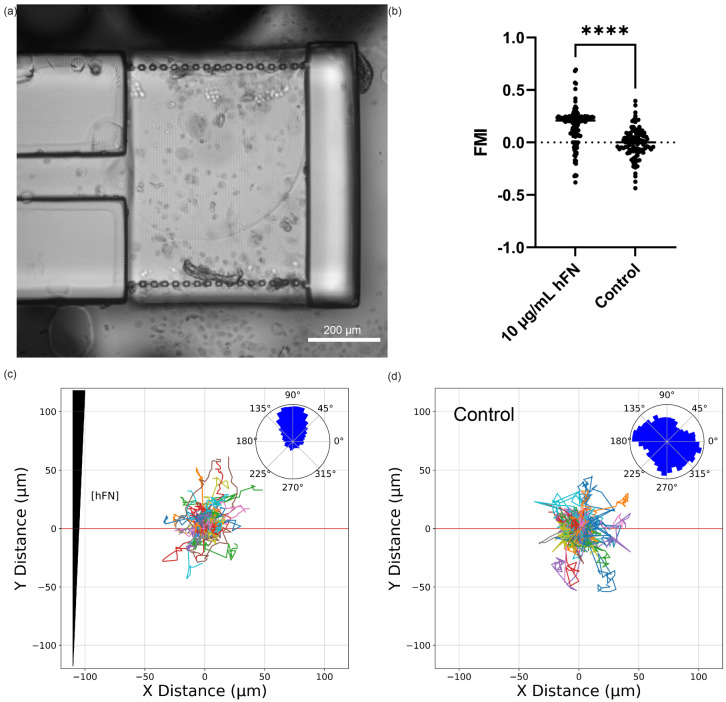
Cell migration in integrated devices with on-chip reservoirs and pumps: (**a**) Microscope image of gradient migration chamber with adhered HLF1 cells. (**b**) Forward Migration Index (FMI) for experiment and control runs, **** *p* < 0.0001. (**c**,**d**) Track plot of cells from gradient migration and control runs, respectively. Corresponding polar histograms are shown as insets.

Figure 7 shows the corresponding experimental results for chemotaxis assays run with the integrated device design. As shown in Figure 7b, HLF1s in the CG experiments migrated towards the 10 μg/mL hFN in the adjacent source channel resulting in an average FMI of 0.16 (*n* = 141 total tracked cells). This value was significantly different (*p* < 0.0001) from the average FMI of −0.005 (*n* = 112 total tracked cells) in the no-CG control experiments. The polar histogram in the inset of Figure 7c shows that the net migration of HLF1 was quite directional towards the hFN in the integrated device. In contrast, the polar histogram in the inset of Figure 7d shows an essentially random movement of cells with no CG.

The primary functional difference between the syringe pump and integrated devices is that the pumping action in the latter is highly pulsatile due to the nature of on-chip 3D-printed pumps compared to off-chip syringe pumps. Nonetheless, the similarity of the results in Figure 6 and Figure 7 clearly demonstrates that this difference is not significant for cell chemotaxis experiments. Our results show that such experiments can be consolidated onto a single chip that does not rely on off-chip fluid sources and flow generation, thereby offering the potential for inexpensive single-chip chemotaxis experiments.

## 4. Conclusions

In this paper, we have demonstrated high-resolution 3D printing of microfluidic features using an avobenzone-based resin formulation through spectral engineering of the 3D printer optical source to ensure that the spectral absorption of the avobenzone fully covers the photopolymerizing source spectrum. With our approach, we have further demonstrated high-resolution 3D printing of pillar arrays, valves, and pumps. The valves have been cycled 1,000,000 times without observable degradation, and the pumps have been characterized. Using these components, we introduced a 3D-printed concentration gradient generator and showed its incorporation in both a syringe pump and fully integrated CG chip design. Cell chemotaxis experiments conducted with both types of designs showed effective and comparable cell migration results. In summary, our generalized 3D printing approach combined with spectral engineering and avobenzone-based PEGDA resin provides an effective biocompatible 3D printing platform for high-resolution cell-based microfluidics.

## Figures and Tables

**Table 1:** Pump states for 5-phase actuation sequence [7]. Each column shows the valve and DC state for a single phase, with red and green denoting closed and open, respectively. The phase interval, Δt, is the time difference between phases, i.e., $\Delta t=t_{i}-t_{i-1}$.

	$t_{0}$	$t_{1}$	$t_{2}$	$t_{3}$	$t_{4}$
$V_{1}$	•	•	•	•	•
DC	•	•	•	•	•
$V_{2}$	•	•	•	•	•

**Table 2:** Pump parameters. All dimensions are in microns.

	Diameter	Control Chamber Height	Membrane Thickness	Fluid Chamber Height
V1	150	40	5	20
DC	300	60	10	25
V2	150	40	5	20

**Table 3:** Pump dimensions (in μm) for integrated chemotaxis assay chip.

	Diameter	Control Chamber Height	Membrane Thickness	Fluid Chamber Height
V1	300	50	20	30
DC	600	50	20	60
V2	300	50	20	30

## Data Availability

The data presented in this study are available on request from the corresponding author.

## Supplementary Materials

The following supporting information can be downloaded at: https://www.mdpi.com/article/10.3390/mi14081589/s1. Figure S1: (a) Endothelial cells seeded on baked PEGDA wells imaged after four hours. Cells seeded on devices baked for three and four days demonstrated the best morphology. No significance was observed between two and five-day bakes. (b) Brightfield images of endothelial cell morphology on a 3D-printed PEGDA device baked for variable durations. (c) Color changes of the UV post-print cured PEGDA throughout the baking process. No significant color change was observed after 1 day of baking. Figure S2: Photomicrographs of the CG region (a) without defocus and (b) with defocus to reduce background pixelation of the CG channel surface as described in the main paper text. Table S1: Rayleigh test values.
