# Supplementary material for: Biocompatible High-Resolution 3D-Printed Microfluidic Devices: Integrated Cell Chemotaxis Demonstration
Source: Micromachines (Basel). 2023 Aug 12;14(8):1589. doi: 10.3390/mi14081589 (PMC10456398; doi:10.3390/mi14081589)
Supplement: Supplementary file 1 [file micromachines-14-01589-s001.zip › micromachines-2533562-supplementary.pdf]

Supplementary Information

Biocompatible high resolution 3D printed microfluidic devices: integrated cell chemotaxis demonstration

Mawla Boaks, Connor Roper, Matthew Viglione, Kent Hooper, Adam T. Woolley, Kenneth A. Christensen, and Gregory P. Nordin

Brigham Young University

S1 Post-print device bake

We investigated post-print baking as a means to improve cell adhesion and survivability. We designed and used a chip containing six wells as shown in Fig. S1. Chips were baked at 80°C in a dry oven for variable intervals. Images of baking devices were taken every day to record changes in device color. After baking, chips were rinsed twice with PBS, and cells were seeded with a density of 100,000 cells/mL in DMEM containing 10% FBS and Pen-Strep. Seeded devices in a humidified incubator were placed in a Petri dish with wet Kimwipes to minimize evaporation. Additionally, media in the wells was replaced every hour. Brightfield images of the cells within the wells were captured approximately four hours after seeding to verify the normal morphology of cells attached to the polymer surface.

We analyzed cell viability on a 3D printed surface after baking at 80°C. We observed a color change in the polymer after baking for one day and minimal color variation in subsequent days. Cell morphology and adhesion to the PEGDA significantly improved after baking the devices for three days or more. The density of spread cells increased from 50 cells/mm^2 to 300 cells/mm^2 by expanding the baking time from two days to three. In trials using devices baked for less than three days, cell attachment was inconsistent as few cells weakly adhered to the polymer but failed to develop adequate morphology. We determined a three-day bake to be the minimum threshold for acceptable cell morphology, as baking time exceeding three days did not significantly increase the observed number of adherent cells. Interestingly, cell morphology in devices baked for five days did not vary significantly from two-day bake devices.

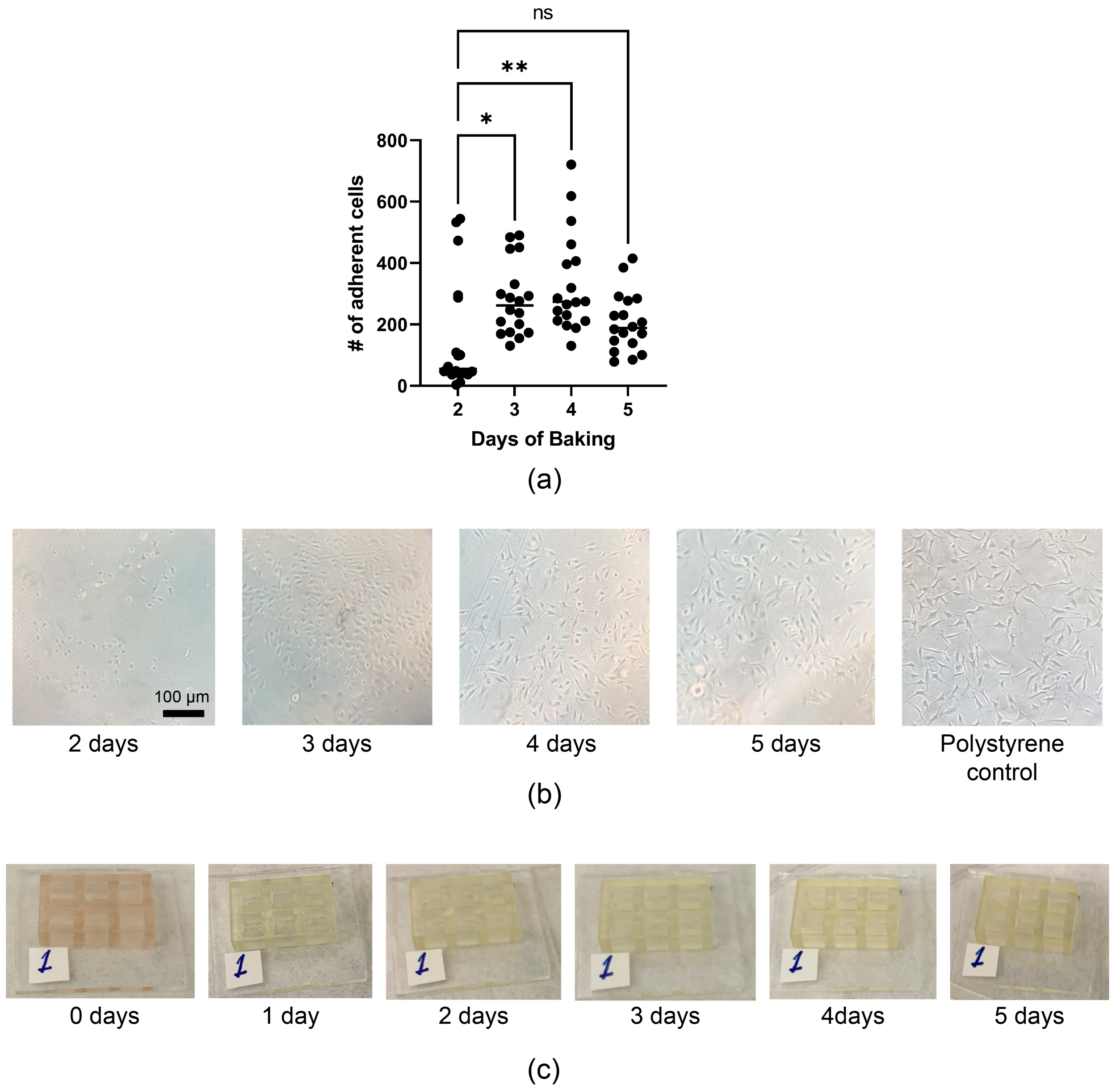

Figure S1: (a) Endothelial cells seeded on baked PEGDA wells imaged after four hours. Cells seeded on devices baked for three and four days demonstrated the best morphology. No significance was observed between two and five-day bakes. (b) Brightfield images of endothelial cell morphology on a 3D-printed PEGDA device baked for variable durations. (c) Color changes of the UV post-print cured PEGDA throughout the baking process. No significant color change was observed after 1 day of baking.

S2 3D printing with deliberate defocus to reduce pixelation

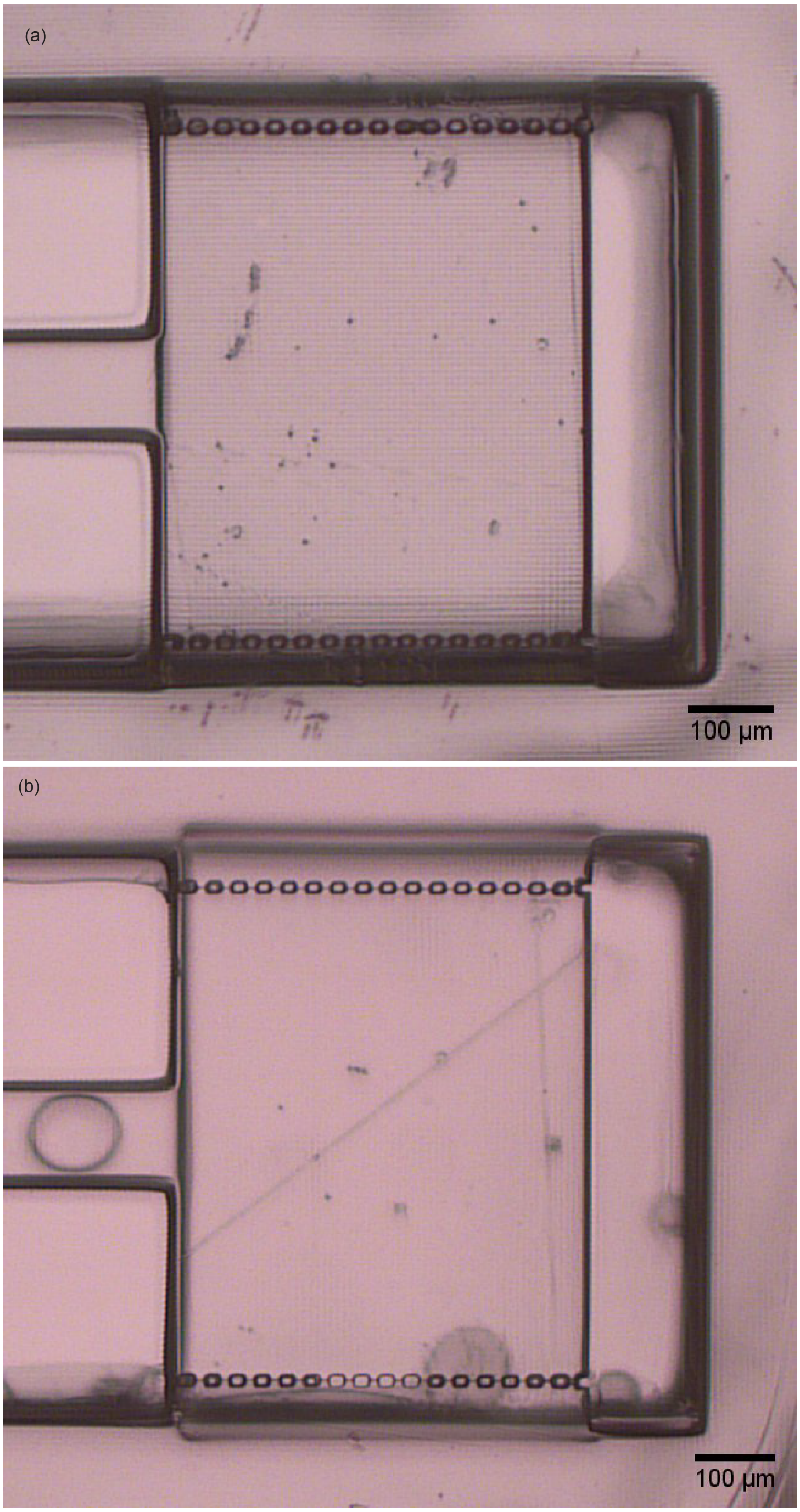

Figure S2: Photomicrographs of the CG region (a) without defocus and (b) with defocus to reduce background pixelation of the CG channel surface as described in the main paper text.

S3 Cell chemotaxis analysis

Device type	Run type	Number of tracks (n)	p Value
Integrated device	Migration Run 1	49	3.61×10^{-5}
	Migration Run 2	30	0.00699473
	Migration Run 3	62	1.36×10^{-7}
	Control Run 1	40	0.377294
	Control Run 2	37	0.63019
	Control Run 3	35	0.19667
Syringe pump device	Migration Run 1	42	0.0231351
	Migration Run 2	56	9.4×10^{-6}
	Migration Run 3	29	1.32×10^{-4}
	Control Run 1	35	0.926111
	Control Run 2	37	0.722626
	Control Run 3	32	0.92447

Table S1: Rayleigh test values
